# Clinical trials in neonates: How to optimise informed consent and decision making? A European Delphi survey of parent representatives and clinicians

**DOI:** 10.1371/journal.pone.0198097

**Published:** 2018-06-13

**Authors:** Virginia Neyro, Valéry Elie, Nicole Thiele, Evelyne Jacqz-Aigrain

**Affiliations:** 1 Department of Paediatric Pharmacology and Pharmacogenetics, Assistance Publique des Hôpitaux de Paris, Robert Debré Hospital, Paris, France; 2 Doctoral School MTCI – Paris Descartes University, Paris, France; 3 European Foundation for the Care of Newborn Infants, EFCNI, Munich, Germany; 4 INSERM Clinical Investigations Center CIC1426, Robert Debré Hospital, Paris, France; 5 EA08 – Paris Diderot University, Paris, France; University of Liverpool, UNITED KINGDOM

## Abstract

**Objectives:**

Parental consent for the participation of their neonate in neonatal research is influenced by the quality of the information delivered and the interaction between parents and investigators. Failure to provide important information may lead to difficulties in the decision making process of parents. This Delphi survey aims to establish a consensus between parent representatives of neonatal associations and healthcare professionals concerning the information deemed essential by both parties in order to improve the recruitment of neonates into clinical trials.

**Method:**

This study was conducted in Europe among parent representatives and healthcare professionals. In this 3-phase study, 96 items were defined by the Scientific Committee (CS), composed of 11 clinicians (from 8 countries) and 1 parent representative of the European network of neonatal associations. Then the Committee of Experts (CE) composed of 16 clinicians were matched by country with 16 national parent representatives and evaluated these items in two rounds. The importance of each item was evaluated by each member of the CE on a scale between 1 and 9 based on their personal experience.

**Results:**

Fifty eight items reached the second and final level of consensus. In contrast to clinicians, parent representatives preferred to be informed about the study by the physician in charge of their child. They also favoured additional support during the informed consent process and stated that both parents need to agree and sign.

**Conclusion:**

The set of 58 items on which parents and clinicians reached consensus will be helpful to healthcare professionals seeking parental consent for the inclusion of a neonate in a clinical trial. Providing parents with information about the trial by the investigator in the presence of the patient’s neonatologist, developing closer contacts with parents and informing them of the available support by parents associations may be helpful for parents.

## Introduction

For years children have been "therapeutic orphans" and denied the benefits of clinical research, most prescribed drugs being unlicensed and/or off-label medicines for this particular population. Since 2007, the European Paediatric Regulation stimulates systematic paediatric drug evaluations through Paediatric Investigation Plans. It aims to improve the development and facilitate accessibility of medicinal products for use in the paediatric population, including in neonates [1]. Clinical trials remain the golden standard for drug evaluation. Whilst in the adult population patients need to provide their consent to participate in a trial, the situation is quite different for neonates, where parents take the responsibility for their child’s enrolment.

Obtaining parental consent relies on providing the necessary information to help parents to take an informed decision. This exchange of information between investigators and parents is a serious challenge for clinicians [2]. Failure to provide important information may lead to difficulties in the decision-making process of parents.

Asking for parental consent in neonatology is complex and several studies have focused on elements that may facilitate communication. The understanding of the randomisation process by parents is particularly important in order to help them decide whether their child should be included in the trial [3,4].

Thus, the optimisation of the quality of information provided to parents is important to help them understand the scientific and ethical rationale of the trial.

Priorities regarding the information that should be provided may differ between parents and investigators [5]. For example, parents prioritise explanations about the current clinical status of their child or the characteristics of the study itself [6]. Understanding the point of view of both parents and clinicians on the type of information to be provided during the informed consent process may result in an increased acceptance and a decrease of withdrawal from studies.

Therefore, this Delphi survey [7–10] aimed to establish a consensus between representatives of parents of neonates and healthcare professionals to identify criteria considered important in the informed consent process.

## Methods

### Ethics statement

This study was evaluated by the ethics committee at Robert Debré Hospital. All participants agreed to participate in this survey by responding positively to an invitation after being informed about the objectives and organisation of the study.

### Purpose, rationale and study design

The purpose of this study was to establish a consensus between parent representatives and healthcare professionals on the information to be provided during the process of obtaining informed consent for the inclusion of a neonate in clinical research.

The rationale for this study was based on the currently very limited literature providing guidance and the observation that the needs of parents and investigators during the informed consent process may differ [5,6].

Using the Delphi methodology this European study was conducted among parent representatives of newborns and healthcare professionals.

The Delphi method consults experts on a specific topic aiming to reach a consensus. It is organised in various rounds during which experts are asked to express their opinion on specific items. Following each round the group’s responses to each item are provided to all participants. Each individual is then encouraged to reconsider and where appropriate change their reply in the next Delphi round, taking into consideration the view of the group. This process is repeated until a consensus is reached.

### Expert panel

This survey included a Scientific Committee (SC) and a Committee of Experts (CE). Experts were people with sufficient practical, political, legal or administrative knowledge of the subject. The impartiality, independence and absence of conflict of interest of participating experts were ensured by the Scientific Committee during the selection process for the Committee of Experts.

#### Scientific Committee—SC

The Scientific Committee—SC (analysts) was responsible for the organisation of the Delphi survey. It selected the members of the Committee of Experts, drafted the successive versions of the questionnaires and analysed and summarised the results after each round. The SC was composed of specialists in the field of neonatology and clinical trials. Members of the SC were selected based on their scientific expertise, years of experience and their number of publications. Furthermore, the SC included the chair of the first pan-European organisation representing the interests of premature babies and their families *(European Foundation for the Care of Newborn Infants—EFCNI)*. The EFCNI has established a network of stakeholders collaborating to improve long-term health of neonates by reducing the rate of preterm births and ensuring optimal treatment, care and support during the neonatal period.

#### Committee of Experts—CE

The Committee of Experts—CE (experts) was composed of healthcare professionals (paediatricians, neonatologists, obstetricians, pharmacologists, researchers, study coordinators, specialists in medical ethics and members of ethics committees) and parents representing the different national organisations of parents of premature babies, collaborating with the EFCNI.

These being themselves parents of neonates born prematurely, they have therefore coped with the complications of such situation. Given their position in the organisations, they transmit discussions, opinions, thoughts and interests of the parents they represent, as well as their own. They were proposed by the central EFCNI chair, Nicole Thiele, because they were considered competent to answer the Delphi questionnaire based on their own experience.

#### Selection of experts and representativeness

The identification and selection of experts was based on criteria recommended by the Delphi methodology [7–12]. The criteria included their willingness and availability to participate in the survey, as well as their qualifications, publications and experience in the field. Further criteria were their interest in the subject matter, their impartiality, independence and absence of conflict of interest. Finally, the diversity of their points of view, geographical location and their scientific expertise were considered.

To ensure representativeness of the responses and to take into account potential country specific particularities a parent representative from the same country was selected for each healthcare professional contributing to the CE.

### Questionnaire preparation and selection of preliminary items

The first version of the questionnaire was drafted after a review of the relevant literature. The search was conducted using PubMed and included the following keywords: informed consent, parents or parental, investigators, clinical trials or paediatric/neonatal trials, children or neonates. Based on this literature review, 92 items, reported as being important during the informed consent process, were included in the first version of the questionnaire and sent to the SC for review.

The SC was asked to keep, remove or modify them as needed, including the possibility to add new items between March and May 2016.

This review resulted in a final questionnaire of 96 items to be evaluated by the CE. None of the members of the CE requested adding further items.

These 96 items were organised into seven headings: I. Information provided to the parents (10 items); II. The information disclosure process (19 items); III. The decision making process (17 items); IV. Criteria that may influence the decision making process (29 items); V. Criteria concerning the informed consent (8 items); VI. Criteria that may encourage a positive decision to the inclusion of the newborn in the study (8 items); VII. Reasons for a negative answer to participation from the parents (5 items).

### Procedure

To facilitate data collection, the questionnaire was developed using an online platform. Data was collected anonymously and kept confidential. Communication not relating to the content of the questionnaire occurred via e-mail.

This Delphi survey was conducted in accordance with the recommendations for the assessment of healthcare quality [10]. Its methodology was consistent with the literature in terms of the number of rounds, number of experts and cut-off levels for consensus. However, the methods of Delphi studies do vary as noted in a systematic review conducted by Boulkedid and colleagues [10] and the “The RAND/UCLA Appropriateness Method User's Manual” [13]. Based on currently used cut-off values, we used a scoring of 1–9 for the importance of the different items with median scores of 7–9 being considered high enough to reach consensus [10,14,15].

**Fig 1:**
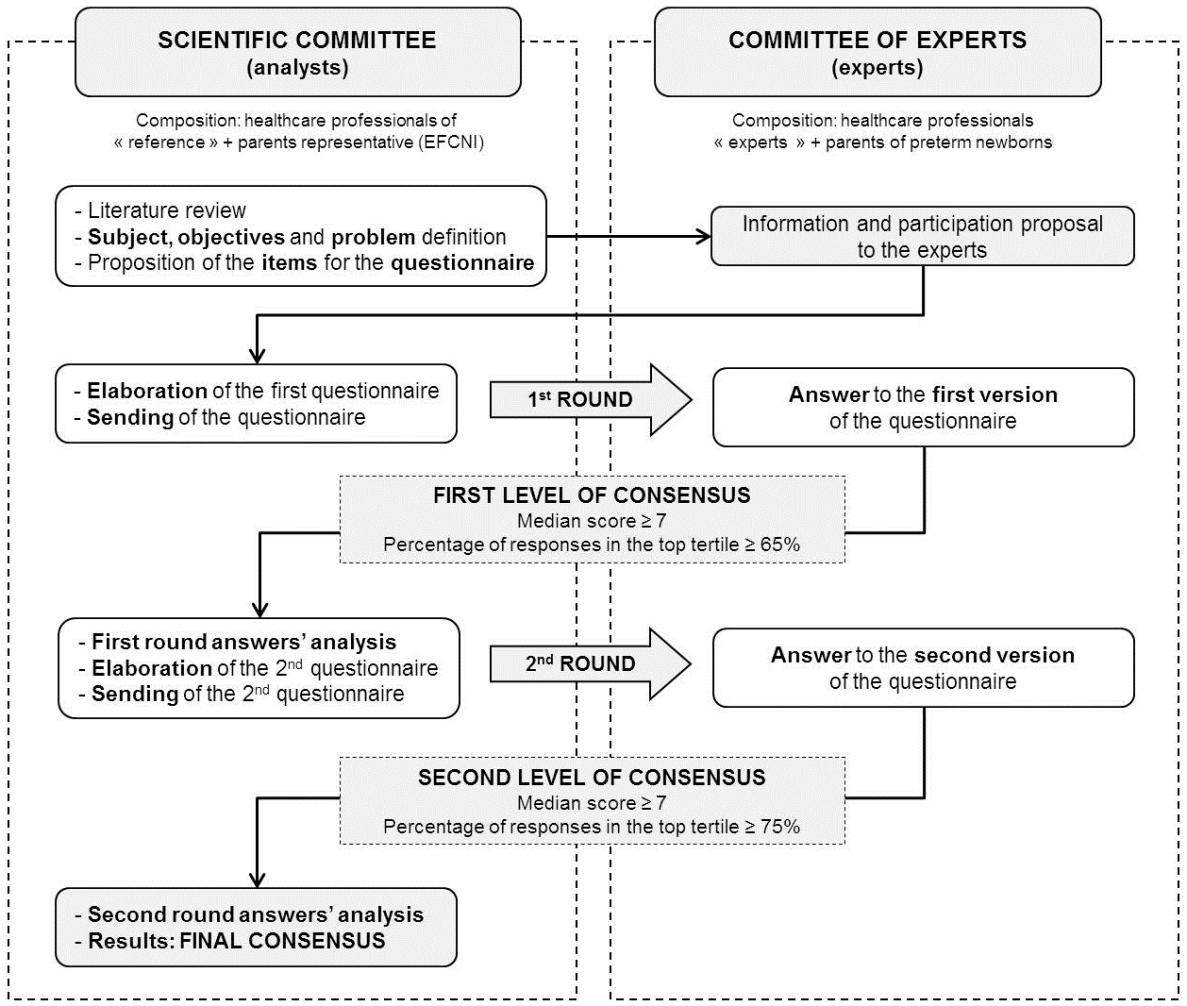
Overall organisation of the Delphi process.

As described in the Delphi methodology, all 96 items were rated twice by the CE (Fig 1). In each round the experts (CE) were asked to rate the items on a scale from 1 to 9 according to the level of importance, based on their personal experience and beliefs: 1 being “not important, not true, should be dropped as an item to consider, totally disagree” and 9 being “extremely important, obligatory, true, must be an item to consider, totally agree”.

#### First round

Round one of the survey was conducted between August 2016 and January 2017. Items with a median score of 7 or higher and rated in the top tertile (7 to 9) by at least 65% of the CE were kept for the second round (first level of consensus). In addition, demographic information on the participants was collected at this occasion (field of expertise, place of employment, function, and knowledge and experience regarding clinical trials).

#### Second round

The second round was performed between February and May 2017. For each item, the CE members were informed of the rating they had provided in the first round as well as a summary of the rating of the CE group for each item (median, percentage of rating in the top tertile and frequency distribution). Finally, items with a median score of 7 or higher and rated 7 or higher by at least 75% of the CE were kept for the final list of items (second level of consensus).

In addition, items and comments not included in the final consensus such as diverging opinions between parent representatives and healthcare professionals were examined and summarised.

## Results

The organisers of this survey invited 25 professionals with several years of experience to become members of the Scientific Committee based on their expertise in the fields of paediatrics, neonatology or clinical research. Five refused to participate due to lack of time or because they considered having insufficient knowledge of the subject matter. A further six did not respond to the request. Two other professionals accepted initially but withdrew in the end. Thus, 12/25 (48.0%) experts from 9 countries composed the Scientific Committee. The group consisted of 11 healthcare professionals and 1 parent representative.

For the Committee of Experts, 26 healthcare professionals were contacted to participate, 10 of which did not respond. Twenty-two national parent representatives were contacted with the help of the EFCNI, 6 of which did not respond. Therefore the Committee of Experts consisted of 16/26 (61.5%) healthcare professionals and 16/22 (72.7%) parent representatives from 10 countries in Europe.

Eleven out of 16 parent representatives (68.8%) declared that they had knowledge of clinical research and 5 (31.3%) had their own child involved in a study. As for clinicians, 15 (93.8%) had previous experience in neonatal trials and 14 (87.5%) had experience in providing information in the context of obtaining informed consent from parents.

Table 1 shows the characteristics of the panellists including their area of expertise.

**Table 1:** Characteristics of the Delphi panellists.

	SCIENTIFIC COMMITTEE	COMMITTEE OF EXPERTS	
		Professionals	Parents
**N**	12	16	16
**Sex**, n (%)			
Female	3 (25.0)	6 (37.5)	11 (68.8)
Male	9 (81.8)	10 (62.5)	5 (31.3)
**Geographic origin**, n (%)			
Belgium	1 (8.3)	2 (12.5)	2 (12.5)
Finland	0	1 (6.3)	1 (6.3)
France	1 (8.3)	3 (18.8)	3 (18.8)
Germany	1 (8.3)	0	0
Hungary	0	2 (12.5)	2 (12.5)
Ireland	0	2 (12.5)	2 (12.5)
Italy	0	1 (6.3)	1 (6.3)
Poland	0	1 (6.3)	1 (6.3)
Spain	2 (16.7)	2 (12.5)	2 (12.5)
Switzerland	1 (8.3)	0	0
The Netherlands	1 (8.3)	1 (6.3)	1 (6.3)
United Kingdom	2 (16.7)	1 (6.3)	1 (6.3)
Canada	2 (16.7)	0	0
United States of America	1 (8.3)	0	0
**Years of experience**, median [range]	32.5 [10–37]*	21.5 [7–41]	-
**Age** (years), median [range]	-	-	42 [27–63]
**Professional setting**, n (%)			
Institutional	12 (100.0)	16 (100.0)	-
Private	0	0	-
**Function and main area of expertise**, n (%)			
Paediatrics	3 (25.0)	3 (25.0)	0
Neonatology	8 (66.7)	8 (66.7)	0
Pharmacology	6 (50.0)	2 (16.7)	0
Clinical trials	11 (91.7)	15 (93.8)	0
Gynaecology	0	1 (6.3)	0
Ethics	0	2 (12.5)	0
Parent association	1 (8.3)	0	16 (100.0)
**Number of children**, median [range]	-	-	2 [1–4]
**Number of sick neonates**, median [range]	-	-	1 [1–3]
**Involvement / experience in providing information to parents of neonates for their inclusion into a clinical trial**, n (%)	11 (100.0)*	14 (87.5)	-
**Involvement of their newborn in a clinical trial**, n (%)	-	-	5 (31.3)
**Responses to questions asked in the questionnaire**			
Are clinical trials in neonates difficult to conduct? YES n (%)	11 (91.7)	13 (81.3)	15 (93.8)
Are neonatal trials required? YES n (%)	12 (100.0)	15 (100.0)**	16 (100.0)
Should they be conducted in "specialized" Neonatal Care Units?YES n (%)	10 (83.3)	10 (66.7)**	12 (75.0)

* Data concerning 11 physicians

** Based on 15 answers

All 32 experts (100%) participated in both the first and second round of the Delphi process.

### First round

**Fig 2:**
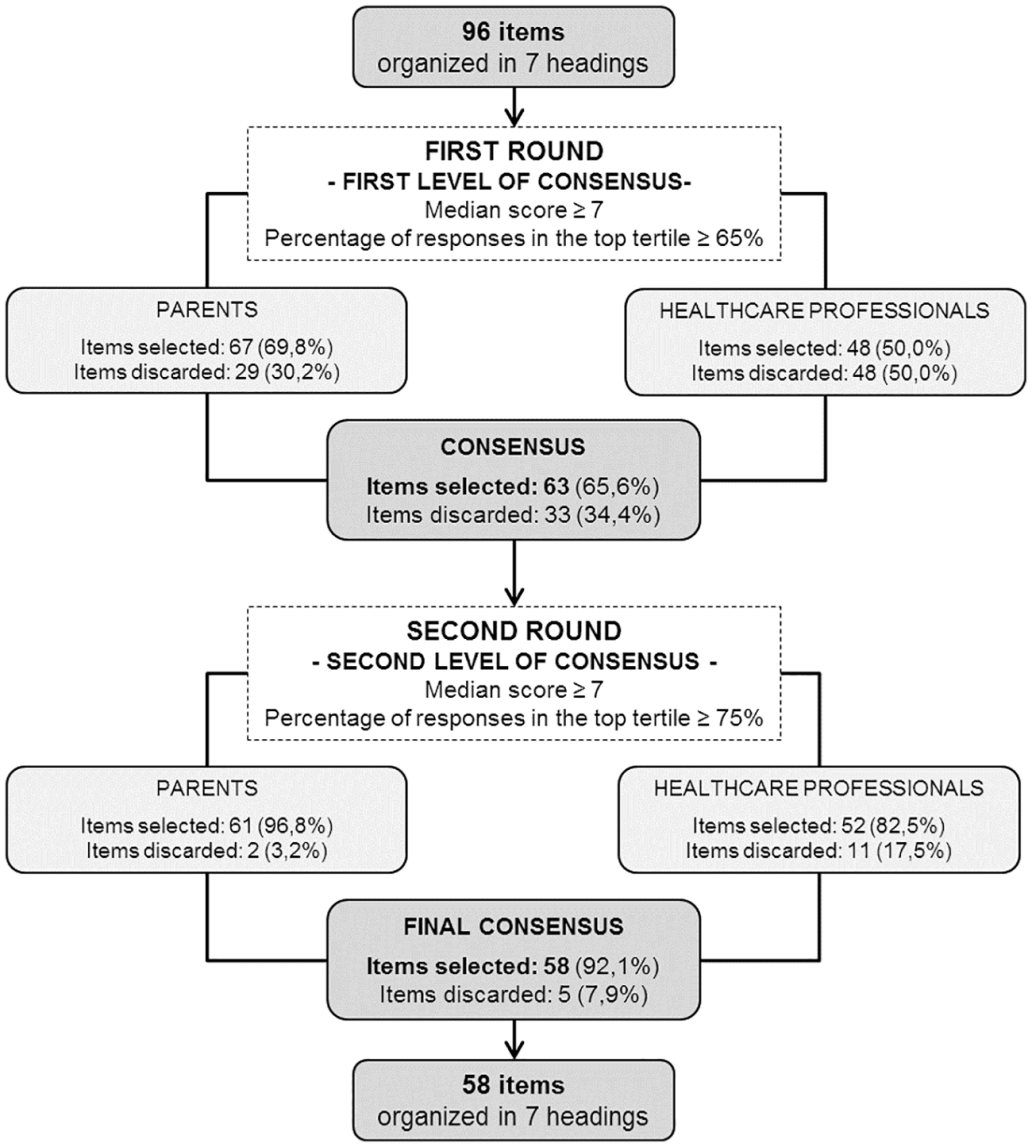
Flowchart of the results of the Delphi survey.

Based on the first level of consensus 63 of 96 items (65.6%) were retained (Fig 2). Thirty-three items (34.4%) were excluded: 18 (18.8%) did not reach a median rating of 7 or above and 15 items (15.6%) were rated in the top tertile but by fewer than 65% of the panellists.

The results of the Delphi survey are presented in Table 2. This includes the median score for each item as well as the percentage of respondents rating the item 7 or higher.

**Table 2:** Results of the Delphi survey.

		**FIRST ROUND**						**SECOND ROUND**						**FINAL SELECTED ITEMS**
		**PROFESSIONALS**		**PARENTS**		**CONSENSUS**		**PROFESSIONALS**		**PARENTS**		**CONSENSUS**		
**SECTION 1: INFORMATION PROVIDED TO THE PARENTS**		Med	% (7–9)	Med	% (7–9)	Med	% (7–9)	Med	% (7–9)	Med	% (7–9)	Med	% (7–9)	
**Information provided to the parents**														
1	Information disclosure sheet and informed consent form should be included in one unique document.	7	56.25	9	87.50	8	71.88	8	62.50	8	93.75	8	78.13	✔
2	Detailed information leaflet and informed consent form should be two separate documents.	3	25.00	5	43.75	4	34.38							
3	Consent can be obtained for only part of the trial (for example: exclusion of samples genotyping or additional investigations: radiography, MRI etc…) if specified in the protocol.	7	62.50	6.5	50.00	7	56.25							
4	All items listed in the information leaflet should be presented orally.	6.5	50.00	9	81.25	8	65.63	8	81.25	8	93.75	8	87.50	✔
5	Only key items listed in the information leaflet should be presented orally.	5.5	50.00	3.5	31.25	4	40.63							
6	The information sheet should be consistent with the oral information given.	9	93.75	9	100.00	9	96.88	9	93.75	9	100.00	9	96.88	✔
7	It is important that the parents have the possibility to read the information sheet before taking the decision.	9	93.75	9	100.00	9	96.88	9	81.25	9	100.00	9	90.63	✔
8	It is important that the parents keep the information sheet and reread it whenever they wish during the trial.	8	93.75	9	100.00	9	96.88	9	93.75	9	100.00	9	96.88	✔
9	Parents who decide to withdraw should be asked whether they give their consent to the use of already collected data.	8	75.00	9	75.00	9	75.00	9	93.75	8.5	87.50	9	90.63	✔
10	Parents who decide to withdraw should be asked if data collected as part of their routine care can still be collected even after withdrawal (i.e. some parents might want to withdraw from an invasive protocol but still accept ongoing collection of data).	7.5	56.25	8	81.25	8	68.75	8.5	93.75	8	87.50	8	90.63	✔
		**FIRST ROUND**						**SECOND ROUND**						**FINAL SELECTED ITEMS**
		**PROFESSIONALS**		**PARENTS**		**CONSENSUS**		**PROFESSIONALS**		**PARENTS**		**CONSENSUS**		
**SECTION 2: THE INFORMATION DISCLOSURE PROCESS**		Med	% (7–9)	Med	% (7–9)	Med	% (7–9)	Med	% (7–9)	Med	% (7–9)	Med	% (7–9)	
**A. Staff responsible for delivery of the information**														
11	The information can be delivered by the physician in charge of the neonate.	7	56.25	8.5	75.00	8	65.63	8	75.00	8	87.50	8	81.25	✔
12	The information can be delivered by a local physician informed of the protocol and study characteristics (not in charge of the neonate).	7	75.00	4	18.75	6	46.88							
13	The information can be delivered by a study investigator (not in charge of the neonate).	7.5	75.00	5.5	37.50	7	56.25							
14	The information can be delivered by the nurse in charge of the neonate.	3	18.75	6.5	50.00	4.5	34.38							
15	The information can be delivered by a research nurse (not in charge of the neonate).	5	43.75	5.5	37.50	5	40.63							
16	All study personnel who provided the information should be able to answer any additional questions or doubts that the parents should have, at any time during the study.	8	87.50	9	100.00	9	93.75	8	87.50	9	93.75	8.5	90.63	✔
17	Contact with the family doctor is recommended, for advice about the trial.	5	31.25	9	62.50	6	46.88							
**B. How to provide information?**														
18	Every reasonable effort should be made so that the information about the study is provided face to face to both parents at the same time.	8	93.75	9	87.50	9	90.63	9	93.75	9	93.75	9	93.75	✔
19	Information about the study can be provided to each of the parents separately.	5	18.75	5	31.25	5	25.00							
20	Information about the study can be provided to only one of the parents, either the mother or the father.	3	0.00	1	12.50	2	6.25							
21	Information about the study can be provided to only one of the parents, either the mother or the father, only if the other parent is not available.	7	75.00	7	62.50	7	68.75	8	87.50	7	56.25	7	71.88	
22	Every reasonable effort should be made so that the information about the study is provided to the other parent, even if it needs to be done by telephone, video, etc.	7	81.25	9	87.50	8	84.38	8	81.25	8.5	93.75	8	87.50	✔
23	Information about the study can be provided, upon request of the parents or as a suggestion of the investigator, in the presence of additional people (family member, general practitioner…)	7	56.25	8	75.00	7	65.63	7	56.25	7.5	87.50	7	71.88	
24	Information about the study should be provided in the native language of the patient’s parents.	8	93.75	9	93.75	9	93.75	8	93.75	9	100.00	9	96.88	✔
25	Information about the study should be provided in the presence of a qualified translator, so that it is provided in the native language of the patient’s parents.	8	75.00	9	100.00	9	87.50	8	81.25	9	100.00	8.5	90.63	✔
26	Information about the study can be provided in a common language that all attendees share and understand.	7	62.50	7	56.25	7	59.38							
27	Information about the study should be provided in words adapted to the level of comprehension of each of the patient’s parents, as NO-technical as practical, so that it remains understandable to them.	9	93.75	9	93.75	9	93.75	9	93.75	9	100.00	9	96.88	✔
28	Identical information has to be provided to both parents.	9	87.50	9	100.00	9	93.75	9	93.75	9	100.00	9	96.88	✔
29	In the context of a neonatal urgent trial or one starting immediately after birth, information can be disclosed to the parents before birth, even if this may result in parental additional stress.	7	81.25	9	56.25	7	68.75	8	87.50	7	75.00	8	81.25	✔
		**FIRST ROUND**						**SECOND ROUND**						**FINAL SELECTED ITEMS**
		**PROFESSIONALS**		**PARENTS**		**CONSENSUS**		**PROFESSIONALS**		**PARENTS**		**CONSENSUS**		
**SECTION 3: THE DECISION MAKING PROCESS**		Med	% (7–9)	Med	% (7–9)	Med	% (7–9)	Med	% (7–9)	Med	% (7–9)	Med	% (7–9)	
**A. When the information is given to parents, please rate some of the identified difficulties**:														
30	The reason why the protocol is conducted is not always well understood.	7	56.25	7	62.50	7	59.38							
31	Parents are not always aware that the protocol is conducted to improve practices and treatment (due to insufficient data available on what the "best practice" is).	7.5	62.50	6.5	50.00	7	56.25							
32	The reason why the protocol is specifically proposed to them is not well understood.	6	37.50	6.5	50.00	6	43.75							
33	The parents are not capable of understanding the different steps that are planned for the neonate during the protocol.	6.5	50.00	5	37.50	5	43.75							
34	The benefits/impact expected for neonates, including their own, are sometimes difficult to understand.	7	56.25	7	62.50	7	59.38							
**B. What help/support is important in the decision making process**														
35	A full confidence in the medical team taking care of the neonate, available to discuss all aspects of the protocol and help with the decision making, might be important in the decision making process.	8	93.75	9	100.00	9	96.88	9	87.50	9	100.00	9	93.75	✔
36	Parental search for information about the protocol or the illness (internet, books, etc), might be important in the decision making process.	6	37.50	8	75.00	7	56.25							
37	External support (from family, friends, etc), might be important in the decision making process.	6.5	50.00	7	68.75	7	59.38							
38	Contacts with specialized parents’ associations, might be important in the decision making process.	6	37.50	8	81.25	7	59.38							
39	Discussions / exchanges with parents who were previously involved in neonatal trials, might be important in the decision making process.	6.5	50.00	8	87.50	8	68.75	7	68.75	8	87.50	7.5	78.13	✔
40	Discussions / exchanges with parents who already accepted to participate to the present trial, might be important in the decision making process.	6	43.75	8	87.50	8	65.63	7	68.75	7	68.75	7	68.75	
41	If necessary, psychological support available during this process, might be important in the decision making process.	6	31.25	8	93.75	7	62.50							
**C. Deadline for positive or negative response to participation in the trial**														
42	Time provided to the parents to make a decision usually depends on the context of the study.	8	100.00	7	87.50	8	93.75	8	93.75	8.5	93.75	8	93.75	✔
43	Time provided to the parents to make a decision may influence it in a positive or negative way.	8	75.00	7	81.25	7.5	78.13	8	75.00	8	100.00	8	87.50	✔
44	Time required by the parents to take a decision should be respected, considering that if they cannot decide within a time line that is reasonable for the researcher or needed for the study, the patient will not be included.	8	87.50	9	81.25	8	84.38	8	93.75	8	93.75	8	93.75	✔
45	Minimum duration of the time provided to the parents to make a decision should be predetermined in the protocol.	4	31.25	8.5	68.75	6.5	50.00							
46	Minimum duration of the time provided to the parents to make a decision should be fixed by the Ethics committee.	2.5	0.00	9	93.75	5.5	46.88							
		**FIRST ROUND**						**SECOND ROUND**						**FINAL SELECTED ITEMS**
		**PROFESSIONALS**		**PARENTS**		**CONSENSUS**		**PROFESSIONALS**		**PARENTS**		**CONSENSUS**		
**SECTION 4: CRITERIA THAT MAY INFLUENCE THE DECISION MAKING PROCESS**		Med	% (7–9)	Med	% (7–9)	Med	% (7–9)	Med	% (7–9)	Med	% (7–9)	Med	% (7–9)	
**A. The neonatal health / situation**														
47	The neonatal health/situation may have an impact on the decision to accept participation.	9	100.00	9	93.75	9	96.88	9	93.75	9	100.00	9	96.88	✔
48	A serious health condition.	8.5	93.75	9	100.00	9	96.88	9	87.50	9	93.75	9	90.63	✔
49	The severity/gravity of the prognostic.	8.5	87.50	9	93.75	9	90.63	9	93.75	9	93.75	9	93.75	✔
50	Limited expected survival.	8.5	81.25	9	93.75	9	87.50	9	87.50	9	100.00	9	93.75	✔
51	Duration of hospital stay prior to the first discussion regarding the trial.	6	37.50	6	43.75	6	40.63							
52	Delay from birth to the first discussion regarding the trial.	7	62.50	6.5	50.00	7	56.25							
53	Anticipated / expected neonatal disease prior to birth.	7.5	62.50	8.5	93.75	8	78.13	8	81.25	8	100.00	8	90.63	✔
54	Maternal / Familial history.	7	62.50	8	68.75	7	65.63	7	56.25	7.5	93.75	7	75.00	✔
**B. Characteristics of the study**														
55	Existence of invasive procedures.	8.5	93.75	9	100.00	9	96.88	9	93.75	9	100.00	9	96.88	✔
56	Expected duration of patient’s participation.	7	56.25	8	87.50	7	71.88	7.5	68.75	8	87.50	8	78.13	✔
57	Expected duration of the study.	7	56.25	7	62.50	7	59.38							
58	Distance between the residence and the center of care.	7	68.75	7	75.00	7	71.88	7	75.00	8	100.00	8	87.50	✔
59	Disruption of the maternal or family life rhythm.	7.5	75.00	9	87.50	8	81.25	8	87.50	8	93.75	8	90.63	✔
60	Need to transfer the patient to another facility.	8	75.00	9	81.25	8.5	78.13	8	93.75	9	93.75	9	93.75	✔
61	Need to extend hospitalisation (even if it is for a limited duration…).	9	81.25	9	87.50	9	84.38	9	87.50	9	93.75	9	90.63	✔
**C. Criteria concerning parents during the decision making process**														
62	Possibility to communicate with other parents in similar situations.	7	62.50	8	81.25	7	71.88	7.5	56.25	7	81.25	7	68.75	
63	Stress caused by the disclosure of information.	7	62.50	7.5	81.25	7	71.88	7	75.00	8	93.75	8	84.38	✔
64	Ability to understand the information received and to reason clearly.	7.5	81.25	9	93.75	9	87.50	8.5	87.50	9	100.00	9	93.75	✔
65	Advice from social services, religious advisors is needed by parents and it may influence the decision making process.	6	31.25	6	50.00	6	40.63							
**D. Decision making process**														
66	Difficulty and importance of making the best decision for the patient.	7	62.50	9	87.50	8	75.00	8	87.50	9	93.75	8	90.63	✔
67	The parents can feel as being entirely responsible for the decision.	7	62.50	9	100.00	8	81.25	8	75.00	8	93.75	8	84.38	✔
68	The professional in charge of information disclosure may influence the decision of the parents.	8	87.50	9	81.25	8	84.38	8	93.75	8	93.75	8	93.75	✔
**E. What do you think are parents expectations regarding the investigators’ skills/competencies?**														
69	Clinical trials expertise.	7.5	75.00	9	100.00	8	87.50	8	87.50	8	93.75	8	90.63	✔
70	Expertise in the medical area where the trial will be conducted.	8	81.25	9	100.00	9	90.63	9	93.75	9	100.00	9	96.88	✔
71	Communication skills.	8	87.50	9	100.00	8	93.75	8.5	81.25	8.5	93.75	8.5	87.50	✔
72	Honesty and sincerity.	9	100.00	9	100.00	9	100.00	9	93.75	9	100.00	9	96.88	✔
73	Transparency of information.	8	87.50	9	100.00	9	93.75	9	93.75	9	100.00	9	96.88	✔
74	Empathy.	8	100.00	9	93.75	9	96.88	9	93.75	9	100.00	9	96.88	✔
75	Availability.	8	87.50	9	100.00	8.5	93.75	8	93.75	9	100.00	8.5	96.88	✔
		**FIRST ROUND**						**SECOND ROUND**						**FINAL SELECTED ITEMS**
		**PROFESSIONALS**		**PARENTS**		**CONSENSUS**		**PROFESSIONALS**		**PARENTS**		**CONSENSUS**		
**SECTION 5: CRITERIA CONCERNING THE INFORMED CONSENT**		Med	% (7–9)	Med	% (7–9)	Med	% (7–9)	Med	% (7–9)	Med	% (7–9)	Med	% (7–9)	
**Signing of the written consent**														
76	Every reasonable effort should be made so that both parents are present at the time of written consent signing.	8	68.75	9	81.25	8	75.00	8	68.75	9	87.50	9	78.13	✔
77	Signing should be required for both parents whatever the situation prior to any trial procedure, unless otherwise specified in the study protocol.	8	62.50	9	87.50	8	75.00	8	68.75	8	87.50	8	78.13	✔
78	Both parents can sign the written consent separately.	8	68.75	9	75.00	8.5	71.88	9	93.75	8	87.50	8	90.63	✔
79	Only one parent may sign if the other is not available (mother still in the maternity ward, neonate transferred from another hospital, father not yet present etc…).	7	68.75	4.5	31.25	6.5	50.00							
80	Only one parent may sign if the other is not available but only in the case of an urgent trial.	6.5	50.00	7	56.25	7	53.13							
81	The signature of the second parent should be sought shortly after the inclusion of the newborn in the trial.	6.5	50.00	8	56.25	7	53.13							
82	Signature of one parent should be enough, generally the mother's, for the participation of the newborn in the study.	3	31.25	1	25.00	2	28.13							
83	Signature of one parent should be enough, and the patient should be able to participate in the study unless the second parent expressly disagrees.	3.5	37.50	1.5	18.75	3	28.13							
		**FIRST ROUND**						**SECOND ROUND**						**FINAL SELECTED ITEMS**
		**PROFESSIONALS**		**PARENTS**		**CONSENSUS**		**PROFESSIONALS**		**PARENTS**		**CONSENSUS**		
**SECTION 6: CRITERIA THAT MAY ENCOURAGE A POSITIVE DECISION TO THE INCLUSION OF THE NEWBORN IN THE STUDY**		Med	% (7–9)	Med	% (7–9)	Med	% (7–9)	Med	% (7–9)	Med	% (7–9)	Med	% (7–9)	
84	An altruistic motivation driven by the existence of collective benefits.	8	81.25	7	56.25	7.5	68.75	8	75.00	8	93.75	8	84.38	✔
85	The possibility to contribute to progress in medicine and medical research.	8	62.50	7.5	87.50	8	75.00	7.5	62.50	8	93.75	8	78.13	✔
86	The need for clinical trials, particularly in neonatology.	7.5	68.75	8	93.75	8	81.25	7	56.25	8	87.50	7	71.88	
87	Promoting a better understanding of one's own child’s disease.	7.5	81.25	8	81.25	8	81.25	8	81.25	8.5	87.50	8	84.38	✔
88	Potential direct and personal benefits for the neonate.	8.5	100.00	9	68.75	9	84.38	8.5	87.50	9	100.00	9	93.75	✔
89	Potential increase of the chance of survival, in the absence of alternatives.	8	93.75	9	75.00	9	84.38	9	93.75	9	100.00	9	96.88	✔
90	Lack of validated existing treatment.	7.5	68.75	9	87.50	8	78.13	8	81.25	8	93.75	8	87.50	✔
91	Potential indirect benefits to other neonates with similar conditions.	8	68.75	7	68.75	7	68.75	8	81.25	8	93.75	8	87.50	✔
		**FIRST ROUND**						**SECOND ROUND**						**FINAL SELECTED ITEMS**
		**PROFESSIONALS**		**PARENTS**		**CONSENSUS**		**PROFESSIONALS**		**PARENTS**		**CONSENSUS**		
**SECTION 7: REASONS FOR A NEGATIVE ANSWER TO PARTICIPATION FROM THE PARENTS**		Med	% (7–9)	Med	% (7–9)	Med	% (7–9)	Med	% (7–9)	Med	% (7–9)	Med	% (7–9)	
92	When involved in a clinical trial, most professionals in charge of information disclosure are not used to the information / consent process.	5.5	31.25	7	75.00	7	53.13							
93	Investigators in charge of the study usually face difficulties to involve other members of the team (physicians, nurses) in the information / consent process to include neonates in research projects.	6	37.50	7	56.25	6	46.88							
94	The involvement of the regular care team (physician, nurse…) is important to create a climate of trust with the parents.	7	62.50	9	93.75	8	78.13	8	87.50	9	87.50	8	87.50	✔
95	The mother is not always available (in the maternity ward), her separation from the neonate may encourage a negative decision.	7	68.75	7.5	68.75	7	68.75	7	87.50	8	81.25	8	84.38	✔
96	It is challenging to add the stress of a trial to the burden of a sick neonate.	8	62.50	8	75.00	8	68.75	8	75.00	8	81.25	8	78.13	✔

### Second round

In the second round, the CE was asked to re-evaluate the 63 items kept following the first round.

Items rated with a median score of 7 or higher and 75% of the respondents rating them in the top tertile were considered for the final consensus. A total of 58 items (92.1%) of the 63 submitted reached this second level of consensus. Five items (7.9%) were excluded.

### Final consensus

The 58 items reaching consensus after the two rounds of the Delphi are presented in Table 3.

**Table 3:** Final consensus of the Delphi survey.

**SECTION 1: INFORMATION PROVIDED TO THE PARENTS**			
**Information provided to the parents**			
1	Information disclosure sheet and informed consent form should be included in one unique document.		
4	All items listed in the information leaflet should be presented orally.		
6	The information sheet should be consistent with the oral information given.		
7	It is important that the parents have the possibility to read the information sheet before taking the decision.		
8	It is important that the parents keep the information sheet and reread it whenever they wish during the trial.		
9	Parents who decide to withdraw should be asked whether they give their consent to the use of already collected data.		
10	Parents who decide to withdraw should be asked if data collected as part of their routine care can still be collected even after withdrawal (i.e. some parents might want to withdraw from an invasive protocol but still accept ongoing collection of data).		
**SECTION 2: THE INFORMATION DISCLOSURE PROCESS**			
**A. Staff responsible for delivery of the information**			
11	The information can be delivered by the physician in charge of the neonate.		
16	All study personnel who provided the information should be able to answer any additional questions or doubts that the parents should have, at any time during the study.		
**B. How to provide information?**			
18	Every reasonable effort should be made so that the information about the study is provided face to face to both parents at the same time.		
22	Every reasonable effort should be made so that the information about the study is provided to the other parent, even if it needs to be done by telephone, video, etc.		
24	Information about the study should be provided in the native language of the patient’s parents.		
25	Information about the study should be provided in the presence of a qualified translator, so that it is provided in the native language of the patient’s parents.		
27	Information about the study should be provided in words adapted to the level of comprehension of each of the patient’s parents, as NO-technical as practical, so that it remains understandable to them.		
28	Identical information has to be provided to both parents.		
29	In the context of a neonatal urgent trial or one starting immediately after birth, information can be disclosed to the parents before birth, even if this may result in parental additional stress.		
**SECTION 3: THE DECISION MAKING PROCESS**			
**A. When the information is given to parents, please rate some of the identified difficulties**			
**B. What help/support is important in the decision making process**			
35	A full confidence in the medical team taking care of the neonate, available to discuss all aspects of the protocol and help with the decision making, might be important in the decision making process.		
39	Discussions / exchanges with parents who were previously involved in neonatal trials, might be important in the decision making process.		
**C. Deadline for positive or negative response to participation in the trial**			
42	Time provided to the parents to make a decision usually depends on the context of the study.		
43	Time provided to the parents to make a decision may influence it in a positive or negative way.		
44	Time required by the parents to take a decision should be respected, considering that if they cannot decide within a time line that is reasonable for the researcher or needed for the study, the patient will not be included.		
**SECTION 4: CRITERIA THAT MAY INFLUENCE THE DECISION MAKING PROCESS**			
**A. The neonatal health / situation**		**E. What do you think are parents expectations regarding the investigators’ skills/competencies?**	
47	The neonatal health/situation may have a an impact on the decision to accept participation.	69	Clinical trials expertise.
48	A serious health condition.	70	Expertise in the medical area where the trial will be conducted.
49	The severity/gravity of the prognostic.	71	Communication skills.
50	Limited expected survival.	72	Honesty and sincerity.
53	Anticipated / expected neonatal disease prior to birth.	73	Transparency of information.
54	Maternal / Familial history.	74	Empathy.
**B. Characteristics of the study**		75	Availability.
55	Existence of invasive procedures.	**SECTION 5: CRITERIA CONCERNING THE INFORMED CONSENT**	
56	Expected duration of patient’s participation.	**Signing of the written consent**	
58	Distance between the residence and the centre of care.	76	Every reasonable effort should be made so that both parents are present at the time of written consent signing.
59	Disruption of the maternal or family life rhythm.	77	Signing should be required for both parents whatever the situation prior to any trial procedure, unless otherwise specified in the study protocol.
60	Need to transfer the patient to another facility.	78	Both parents can sign the written consent separately.
61	Need to extend hospitalisation (even if it is for a limited duration…).	**SECTION 6: CRITERIA THAT MAY ENCOURAGE A POSITIVE DECISION TO THE INCLUSION OF THE NEWBORN IN THE STUDY**	
**C. Criteria concerning parents during the decision making process**		84	An altruistic motivation driven by the existence of collective benefits.
63	Stress caused by the disclosure of information.	85	The possibility to contribute to progress in medicine and medical research.
64	Ability to understand the information received and to reason clearly.	87	Promoting a better understanding of one's own child’s disease.
**D. Decision making process**		88	Potential direct and personal benefits for the neonate.
66	Difficulty and importance of making the best decision for the patient.	89	Potential increase of the chance of survival, in the absence of alternatives.
67	The parents can feel as being entirely responsible for the decision.	90	Lack of validated existing treatment.
68	The professional in charge of information disclosure may influence the decision of the parents.	91	Potential indirect benefits to other neonates with similar conditions.
**SECTION 7: REASONS FOR A NEGATIVE ANSWER TO PARTICIPATION FROM THE PARENTS**			
94	The involvement of the regular care team (physician, nurse…) is important to create a climate of trust with the parents.		
95	The mother is not always available (in the maternity ward), her separation from the neonate may encourage a negative decision.		
96	It is challenging to add the stress of a trial to the burden of a sick neonate.		

After the evaluation of the CE, all of the 7 initial headings were maintained: I. Information provided to the parents (7/10 items); II. The information disclosure process (9/19 items); III. The decision making process (5/17 items); IV. Criteria that may influence the decision making process (24/29 items); V. Criteria concerning the informed consent (3/8 items); VI. Criteria that may encourage a positive decision to the inclusion of the newborn in the study (7/8 items); VII. Reasons for a negative answer to participation from the parents (3/5 items).

### Differences between clinicians and parent representatives

During the first round parent representatives and healthcare professional agreed on the assessment of 65 out of 96 items (level of agreement: 67.7%). However, 31 items (32.3%) were rated differently between the two groups. Parent representatives rated 25 items as important (median score 7 or higher and rated 7–9 by at least 65% of respondents) but healthcare professionals did not agree. In turn 6 items were rated as important by healthcare professionals but parent representatives did not agree.

Selected items were distributed differently among parent representatives and healthcare professionals. Only 48 items (50.0%) reached the first level of consensus when rated by the healthcare professionals, whereas 67 (69.8%) would have been retained by parent representatives (Fig 2).

In the second round, healthcare professionals and parent representatives agreed on the evaluation of 52 out of 63 items (level of agreement: 82.5%). The remaining 11 items (17.5%) were rated differently by the two groups of experts. Parent representatives assessed 10 items as important but healthcare professionals did not agree. One item was considered important by healthcare professionals but parent representatives did not agree.

Similar to the differences observed in the first round, parent representatives and healthcare professionals differed on the number of items they selected as being important in the second round. Parent representatives considered 61 out of the 63 items (96.8%) as being important whilst healthcare professionals chose 52 items (82.5%).

## Discussion

The present Delphi survey was conducted in order to obtain a consensus between parent representatives and healthcare professionals on the information disclosure process to parents for the inclusion of neonates in clinical trials.

The inclusion of subjects in neonatal research has proved to be a problematic process. Obtaining a valid informed consent for the inclusion of a newborn in a clinical trial depends on the proxy consent given by the parents [16]. In general, consent for participation in clinical research is considered valid if the following three conditions have been fulfilled: information, comprehension and voluntariness [17]. However for a sick neonate, all three conditions may be difficult to assess [18]. First of all, the provision of complete and accurate information about the trial to parents depends mostly on the training and skills of the staff communicating with the parents. Effective communication during the informed consent process is not only important in the context of clinical trials in neonates but in any trial including oncology trials [19,20]. Furthermore, parental comprehension may be compromised by their level of medical understanding, the urgency of the trial, emotional distress and many other factors such as the quality of the written and oral information provided [19]. Finally, the free will of a parental decision may not be easy to assess in situations where parents are vulnerable and they might be at risk of being influenced by someone else’s opinions or beliefs.

Informed consent in neonatology is the result of a conglomerate of circumstances both relative to the clinicians in charge of the study and the subjects expected to give authorization for the newborn’s participation. Basic elements to be included in a consent form, such as the purpose and procedures of the study, anticipated risks or potential benefits are described in the Guidelines for Good Clinical Practice (GCP) [21], and later confirmed using the Delphi method to develop a guideline for clinical trial protocol content [22].

Nevertheless, the process of providing information in order to help with the decision of whether or not to participate in a clinical trial remains to be explored further. Understanding the reasons resulting in an affirmative or negative parental decision to include their newborn in a trial may also help improve the informed consent process.

A multidisciplinary panel of 32 experts including both healthcare professionals in the field of neonatal/paediatric clinical research, as well as parent representatives evaluated a total of 96 items divided in 7 sections concerning this complex informed consent process.

The questionnaire was organised in a way that allowed exploring what information needs to be delivered to parents and by whom (sections 1 and 2); what the decision making process may represent for them (section 3); which criteria may influence their decision in a positive or negative way (headings 4, 6 and 7); and finally, their expectations concerning the signature of the informed consent (section 5).

Parent representatives and healthcare professionals agreed on the rating of most items concerning the delivery of information. However, on some items, the two groups shared different points of view which appeared to be influenced by their personal or professional experience.

The information presented orally should be as complete as possible, consistent with the items included in the information leaflet and be provided in the native language of the parents, which may require the help of a qualified translator. As described in the GCP guideline, the words used to describe the trial and its characteristics should be non-technical and understandable for lay people [21]. Information should be provided to both parents at the same time. However, clinicians agreed, in both Delphi rounds, that information could be given to only one of the parent if the other was not available. However, they noted that the other parent should be informed as soon as possible. Parent representatives did not agree with the suggestion that only one parent could be informed. This resulted in the removal of this item at the end of the first round. Parent representatives were also in favour of having a third person present during the informed consent process, such as a family member or their family doctor. However, healthcare professionals did not share this view.

Furthermore, parent representatives expected information on the trial to be provided by the physician in charge of the child. In contrast, healthcare professionals considered, in agreement with article 4.8.5 of the ICH Guideline for GCP, that information should be given by a professional not in charge of the patient such as the investigator or another physician informed of the protocol [21].

When considering the timing for the informed consent process parent representatives initially considered that information should not be given before the child is born. This view included trials beginning immediately after birth and emergency treatments. This observation contradicted the suggestions made by parents in a survey conducted in a Canadian neonatal intensive care unit (NICU). When asked about improvements to be made in the recruitment process, parents recommended that information about the study should be provided before birth to allow sufficient time for reflection [23]. The time the parents have to make a decision may vary between studies. Insufficient time adds considerable stress to parents who find themselves already in a difficult situation. It is also a factor that may influence the decision making process negatively and could make the consent provided not valid [24,25]. Parent representatives responding to this Delphi survey requested that the minimum duration of time provided to make a decision should be predetermined in the protocol and established by an ethics committee. Currently available guidelines present insufficient information about the appropriate time point and how much reflection time should be allowed for seeking parental consent[26]. Studies about continuous consent have suggested the usefulness of providing information to research participants at different stages of a trial in order to help clinicians obtain optimal informed consent [27].

Surprisingly, the members of the Committee of Experts considered none of the 5 items describing potential difficulties encountered by the parents when information about the trial is provided to them as being important. Neither healthcare professionals nor parent representatives thought that misunderstanding the purpose of the study and its potential impact could influence the decision making process. Most of the experts consulted for this survey had sufficient knowledge about clinical research therefore they might be less aware of the challenges faced by parents unfamiliar with clinical trials. However, different studies seeking the points of view of parents showed that understanding of the characteristics of a clinical trial was not always sufficient [28,29]. Some parents showed no or insufficient recollection of giving consent for participation in a research project. Trial related procedures and treatments were considered by some parents as part of routine medical care [30]. Interestingly, even if the level of understanding by parents was not complete, they showed satisfaction about the information given by the investigators [29]. This may explain why a lack of understanding of certain medical terms or specific criteria does not necessarily compromise the whole decision making process.

Parent representatives and healthcare professionals disagreed on items of support that may be useful for the parents. Parent representatives considered seeking themselves information about the study to be helpful, as well as having support from friends and members of the family. They strongly agreed that contact with specialised parents associations or psychological assistance might be important during the decision making process. However, it has to be noted that these parents are members of such specialised parents associations. By contrast, healthcare professionals did not consider these items to influence the decision making process of parents. However, a previous Delphi survey reported the importance of having additional support, such as the possibility to exchange with parents previously involved in neonatal research [31]. As expected, trusting the members of the medical team responsible for the care of the newborn is considered essential by parents facilitating the decision making process [32]. The whole CE agreed that reliability and availability of the clinicians were important. Other skills parent representatives expected from investigators are clinical research expertise as well as knowledge of the medical speciality concerned by the study. Parents trust the information provided if investigators have good communication skills and are honest, sincere and clear when informing about a trial. The availability of professionals at any time of the decision making process is considered fundamental by parents.

Many different criteria can influence the decision making process and parents being more or less stressed and vulnerable during the informed consent process. The health status of the newborn including the seriousness of the condition and prognosis can influence the attitude of parents. Parent representatives attached more importance than professionals, in terms of item rating, to the possibility of being aware of a neonatal disease prior to birth giving them more time to think about treatment strategies or potential inclusion in a research.

Both groups of experts agreed in both rounds that some study characteristics such as invasive procedures and the disruption of family life if the child is transferred to another facility may influence the decision making process. The need to prolong the duration of hospitalisation, as well as the duration of trial participation was considered important by all experts.

Parent representatives considered it important that parents facing a request to include their newborn into a clinical trial should have the opportunity to talk to parents who have been in a similar situation. In particular, considering that having a neonate who is critically ill is already very stressful for parents. However, clinicians did not agree with this view.

This survey confirms that the responsibility of taking the decision to include their newborn in clinical research can be overwhelming for parents. Parents felt strongly about the importance of making the best decision for their child and considered being fully responsible for that choice. They may feel that they do not have a full understanding of all the factors to be considered and their implications whilst being the only ones who can decide what is best for their child [33,34]. In both rounds all of the experts agreed that the professional responsible for providing the information to the parents may influence the opinion of the parents about having their child included into the trial.

Final consensus was obtained for nearly all proposed items relating to reasons that might encourage a positive decision of including a neonate into a clinical trial. Of course, the possibilities of increasing the chances of survival or having other direct benefits for the neonate are a strong motivation for the acceptance to enter a child into a trial. Similarly, a proposal to study a drug is more easily accepted when there is a lack of established treatments. Potential direct or indirect benefits for other neonates in similar situations, as well as the contribution to the understanding of the child’s disease also played a role in encouraging acceptance of the inclusion of the newborn [35]. Parent representatives strongly agreed that contributing to the progress in medicine and neonatal research encouraged a positive decision, whereas clinicians considered that it would not motivate parents. Interestingly, all healthcare professionals agreed that personal and direct benefits for the patient would be the most important point for parents to include their child into a trial. However, during the first round, this was certainly not the most important item for parent representatives.

Parents who agree to have their child participate in a clinical trial need to sign the informed consent form provided by the investigator. All experts agreed that both parents should be present at the time of signing the informed consent form. However, clinicians rated this lower than parent representatives. Both signatures are required to officially begin participation of the neonate to the trial, but these can be obtained separately, as reported by our Committee of Experts. Whilst clinicians considered it acceptable that just one parent signs, parent representatives felt that both parents need to sign even in countries where legally only one signature is considered sufficient. Despite efforts made to standardise paediatric clinical research in Europe, requirements for obtaining consent depend on the different national laws and regulations and therefore vary widely between countries [36]. The members of our panel of Experts come from 10 different European countries. Six of them (Hungary, Finland, Ireland, Poland, Spain and United Kingdom) require only one parent to sign. The other 4 (Belgium, France, Italy and the Netherlands) demand, in most situations, both signatures at some point in the process [37].

This is, to our knowledge, the first European consensus obtained among healthcare professionals and parent representatives on the information disclosure process and request of written consent for the inclusion of neonates into a clinical trial. As expected, this Delphi survey has revealed various important issues to be taken into consideration in neonatal research with some of the items reaching final consensus and others showing differences between parent representatives and physicians.

It should be pointed out, as a limitation, that only 5 out of the 16 parent representatives in the Committee of Experts had themselves experienced the inclusion of their newborn into a clinical trial. Nevertheless, all of them having had preterm infants, these parents had experienced the difficulties linked to the hospitalisation of a neonate. Seeking their advice on the informed consent process is therefore justified. The outcome of the survey might have been different if members of the Committee of Experts would have requested to comment or modify the survey items prior to the first round. The final consensus obtained here concerned the items reaching the pre-established level of agreement for all members of the Committee of Experts but it does not necessarily reflect the individual views of parent representatives or clinicians.

In conclusion, the news that their newborn is seriously ill can be devastating and stressful for parents. For some trials beginning immediately after birth, the time parents have for making a decision about consent is often very limited and the weight of the decision weighs on them. Every effort should be made by the investigators in charge of the study, as well as the medical team, to improve the circumstances of providing information during the informed consent process. It is important that the expectations of parents are well understood and that support and help is provided to facilitate a truly informed consent process.
